# High-efficiency and stable piezo-phototronic organic perovskite solar cell

**DOI:** 10.1039/c8ra00520f

**Published:** 2018-02-27

**Authors:** Ke Gu, Dongqi Zheng, Lijie Li, Yan Zhang

**Affiliations:** School of Electronic Science and Engineering, School of Physics, School of Physical Electronics, University of Electronic Science and Technology of China Chengdu 610054 China; Multidisciplinary Nanotechnology Centre, College of Engineering, Swansea University Swansea SA1 8EN UK L.Li@swansea.ac.uk; Beijing Institute of Nanoenergy and Nanosystems, Chinese Academy of Sciences Beijing 100083 China zhangyan@uestc.edu.cn; College of Nanoscience and Technology, University of Chinese Academy of Sciences Beijing 100049 China

## Abstract

Perovskite materials are regarded as next-generation organic photovoltaic (OPV) materials due to their excellent physical and chemical properties. Recent theoretical and experimental advances also revealed the piezoelectric properties of CH_3_NH_3_PbI_3_ perovskite thin films. In this work, a CH_3_NH_3_PbI_3_ perovskite piezo-phototronic solar cell is studied in theory. The output parameters such as open circuit voltage, current–voltage characteristics, fill factor, power conversion efficiency, and maximum output power with external strains are presented. The coefficient to characterize piezo-phototronic modulation is also calculated for the piezo-phototronic solar cell. With the change of strain, the output performance can be controlled and enhanced. This principle can offer not only a novel and unique approach to produce high-performance, stable perovskite solar cells, but also a principle to design new piezoelectric perovskite optoelectronic devices.

## Abstract 2

Enhancing the performance of perovskite solar cells with strain based on a piezo-phototronic effect.

## Graphical abstract

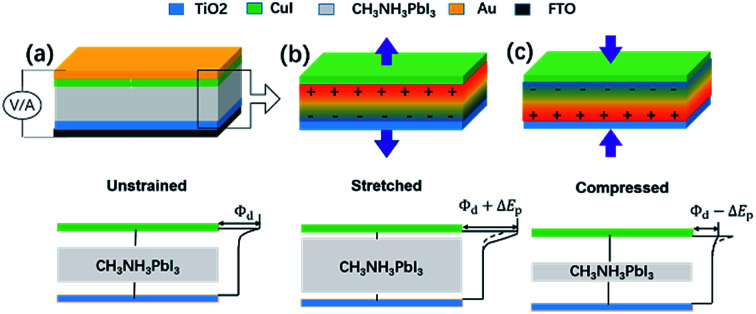

## 1. Introduction

Organic photovoltaic (OPV) devices have been leading the research into energy science and technology.^1–3^ In recent years, much attention has been devoted to the perovskite materials and their applications in solar energy conversion because of their high absorbance, low carrier recombination rate, outstanding ferroelectric properties and easier synthesis.^4–6^ Among them, perovskite solar cells (PSC) with CH_3_NH_3_PbX_3_ perovskites (X = Cl, Pb, and I) as absorber layers show great potential in fabricating low-cost and large-scale commercial solar cells, whose efficiency has increased from 3.8% to over 22% in the past five years.^7–10^

The concept of piezoelectric enhanced performance of nanostructure semiconductor device were put forward in 2007, promoting the interdisciplinary development in the field of nanotechnology.^11^ Combining piezoelectric and semiconductor properties, piezoelectric semiconductor materials such as ZnO, GaN, CdS have been used to fabricate multifunctional stretchable semiconductor devices, such as flexible photodetectors,^12^ piezophototronic light emitting diodes^13^ and piezo-nanogenerator.^14^ Besides, piezo-phototronic and pyro-phototronic effect also enables the improvement in power conversion efficiency of low-dimensional solar cells.^15–18^

Recent breakthrough in photovoltaic devices has developed a stable and durable CH_3_NH_3_PbI_3_ perovskite solar cell with a stable efficiency of 8%, which overcomes the problem of PSC's application in the ambient environment.^19^ CH_3_NH_3_PbI_3_ thin films have the piezoelectric properties in recent experiments.^20^ Piezoelectric materials have been used to design energy harvesting devices, such as piezoelectric generator.^21^

From semiconductor physics point of view, light management and carrier management are two critical factors determining the performance of solar cells.^22^ The basic principle of a solar cell is that photo-generated electron–hole pairs are separated on the interface of metal–semiconductor or p–n junction. In piezo-phototronic solar cell, piezoelectric charges increase the built-in electric field, which can efficiently enhance the separation of electron–hole pairs and improve the carrier management.^15^ The piezo-phototronic perovskite solar cells can be a promising candidate for flexible OPV devices. There are several newly discovered semiconductor properties of CH_3_NH_3_Pb_3_I_3_. The large piezoelectric constant (0.83 C m^−2^) and the small relative dielectric constant (32) of the perovskite materials result in higher current density and higher adjustability. The adjustability shows the higher modulation of output parameters including open-circuit voltage, output power density, fill factor, PCE as well as the regulator further presented. Besides, the stable and durable CH_3_NH_3_PbI_3_ also demonstrates perfect output characteristics with short circuit current density as 14 mA cm^−2^ and open circuit voltage as 0.45 V, comparable to other novel solar energy conversions, which sets the foundation for the high-performance PPSC.^19^

**Fig. 1:**
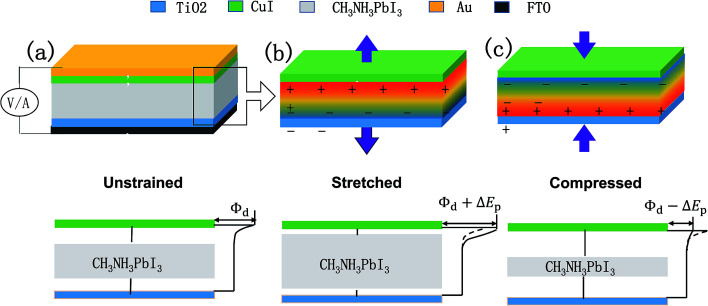
(a) Schematic and energy band diagram of a piezo-phototronic perovskite solar cell. (b) Schematics and energy band diagram of the PPSC with tensile strain applied. (c) Schematics and energy band diagram of the PPSC with compressive strain applied. The transitional color indicates the distribution of the piezo-potential on the CH_3_NH_3_PbI_3_ thin film.

This work provides a prototype of the perovskite piezo-phototronic solar cell, which has improved performances by applying piezoelectric and photovoltaic properties of CH_3_NH_3_PbI_3_ thin films. A schematic of the device architecture is shown in Fig. 1. The main structure is the perovskite material sandwiched by CuI (as HTM) and TiO_2_ (as ETM). The upper layer is an Au electrode, while the base is the PET substrate. Here, we treat the interface between the top layer and the layer beneath as metal–semiconductor (M–S) contact, and the bottom contact (between the base layer and the layer on its top) as ohmic contact. When compressive and tensile strains are applied on the CH_3_NH_3_PbI_3_ layer, the piezoelectric polarization charges are induced at the contact. Fig. 1(b) and (c) show the modulation role in the Schottky barrier played by piezoelectric field, which can increase or decrease the barrier respectively. The characteristic parameters to measure the performance of solar cells, such as current–voltage characteristics, open circuit voltage, maximum output power, fill factor, and power conversion efficiency are studied. The principle of piezo-phototronic solar cell can offer a unique and novel approach to produce an organic photovoltaic cell with stability, higher efficiency and lower cost.

## 2. Piezo-phototronic modulation on PPSC

Previous research works have elucidated the characteristics of piezo-phototronic solar cell by semiconductor physics and piezoelectric theory. Here, the detailed PPSC theoretical analysis is presented combining the physics-based analytical model for perovskite solar cells^23^ and piezo-phototronic theory. Given the extraordinarily long diffusion length,^24,25^ the recombination in the perovskite layer is ignored, so the analytical model of diffusion and drift of photo-generated carriers can be derived as:1
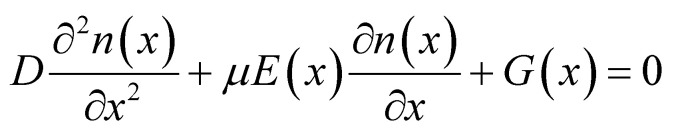
2
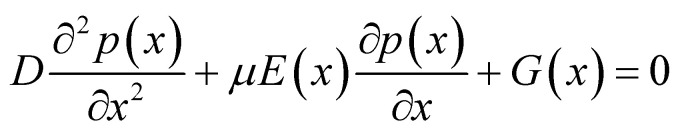
where *n*/*p* is the electron/hole concentration, *D* and *μ* are the diffusion coefficient and mobility, respectively, and *G*(*x*) represents photogeneration varying with the position *x*. *E*(*x*) is the electric field with the perovskite layer determined by the position.

Then the detailed current–voltage characteristics can be expressed as:3
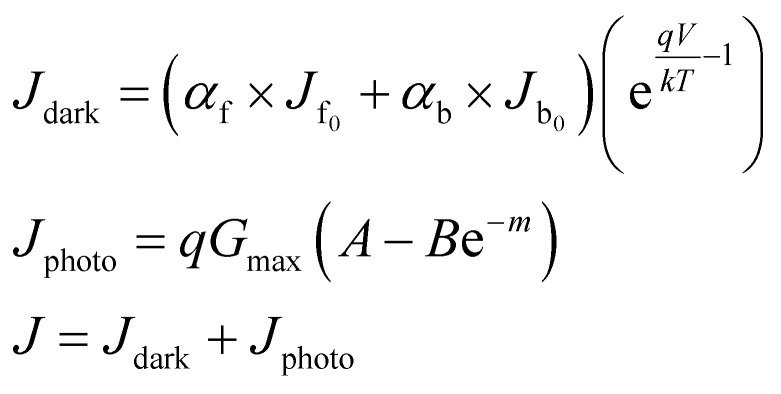
where *A*, *B*, *α*, *m* are the functions of *t*_0_ (the thickness of the perovskite layer), *J*_f_0__ and *J*_b_0__ are the dark diode current at the transport layer, *V*_bi_ is the built-in potential across the perovskite layer, *D* is the diffusion coefficient, *s* is the effective surface recombination velocity, *W*_d_ is the equilibrium depletion width, *G*_max_ is the integration of the position-dependent photon absorption by calculating the transfer matrix.

Based on piezo-phototronic theory, the current density of piezo-phototronic solar cell is given by:4
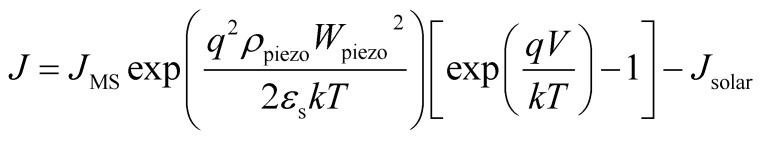
where *J*_MS_ is the saturation current density at the MS interface, *ρ*_piezo_ expresses the density of the piezoelectric charges, *W*_piezo_ is the width of charges distribution, *k* is the Boltzmann constant, *T* denotes the temperature, *ε*_s_ represents the dielectric constant, *J*_solar_ is the short circuit current density, and *V* stands for the applied voltage.

The open circuit voltage can be expressed as:5
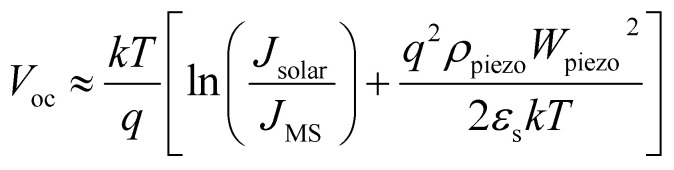
where *γ* represents the scale ratio which depicts piezo-phototronic modulation for open circuit voltage and output characteristics of piezo-phototronic solar cell:6
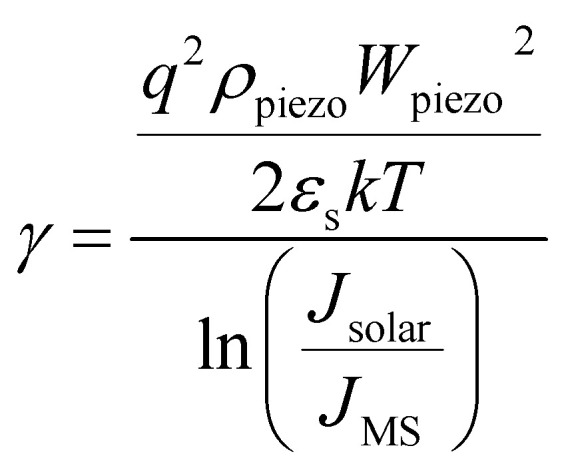


In solar cell physics, the fill factor is the ratio of the maximum output power to the product of open circuit voltage and short circuit current, and can be obtained by:7
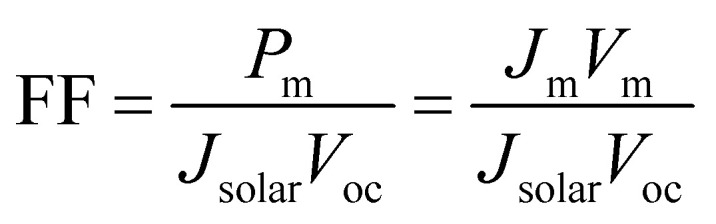


Fill factor can be influenced when the generation of charges changes significantly between open-circuit and short-circuit conditions. In the classical drift-diffusion model, recombination process which depends on a complex interplay between the effects of thickness,^26^ charge transport, recombination strength and light intensity plays an important role in the loss of photo-generated pairs.^27^ However, the extraordinarily long diffusion length^25^ in perovskite materials is larger than the active layer, thus the effect of recombination is neglected in this work.

The power conversion efficiency (PCE) is the ratio of maximum output power to the total incident power, which can evaluate the overall efficiency of the solar cell. The PCE can be obtained as:8
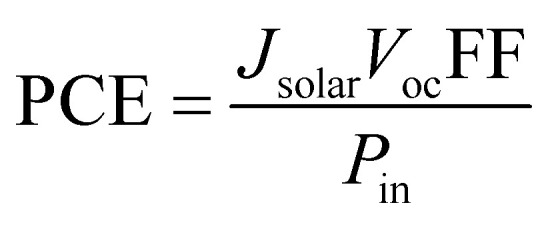


## 3. Results and discussion

Typical parameters and coefficients are used to calculate the open circuit voltage, maximum output power, power efficiency, and fill factor. The temperature is set to 300 K, the width of piezo-charges distribution *W*_piezo_ is 54.3 nm,^28^ the relative dielectric constant is 32. The piezoelectric constant of polar configuration of single crystal CH_3_NH_3_PbI_3_ which has molecular dipoles aligned preferentially along *c*-axis while distributed isotopically within the *ab* plane is used in the calculation and the piezoelectric constant along *c*-axis for CH_3_NH_3_PbI_3_ is 0.83 C m^−2^.^20^

**Fig. 2:**
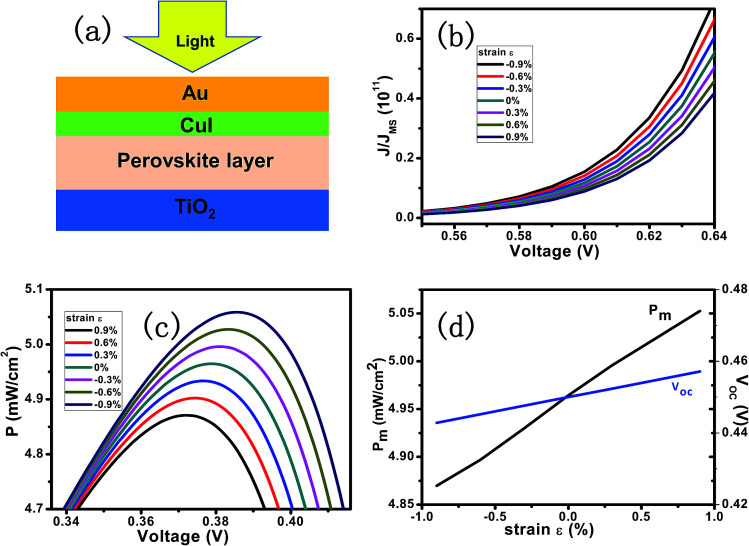
(a) Schematic of a PPSC with CH_3_NH_3_PbI_3_ thin film sandwiched between CuI and TiO_2_. (b) Relative current density varying with voltage when applied compressive strains ranging from −0.9% to 0.9% applied. (c) Output power of a PPSC as a function of voltage with various strains applied. (d) Maximum output power and open-circuit voltage under various compressive strains.

Fig. 2(a) presents the calculation model. A layer of CH_3_NH_3_PbI_3_ is sandwiched between upper HTM and bottom TiO_2_ composite layer. The *J*–*V* characteristics of the PPSC with externally applied strain ranging from −1% to 1% at a short circuit current density of 14 mA cm^−2^ is shown in Fig. 2(b). Fig. 2(c) shows the relation between the output power and voltage, which means the current density increases proportionally to applied strains, and peaks at *V*_m_. By solving the above equations, the *V*_oc_ and *P*_m_ as a linear function of the strain are shown respectively in the Fig. 2(d). The introduction of the piezo-phototronic effect improves the performance of the PPSC evidenced by the increased *V*_m_ and *P*_m_.

**Fig. 3:**
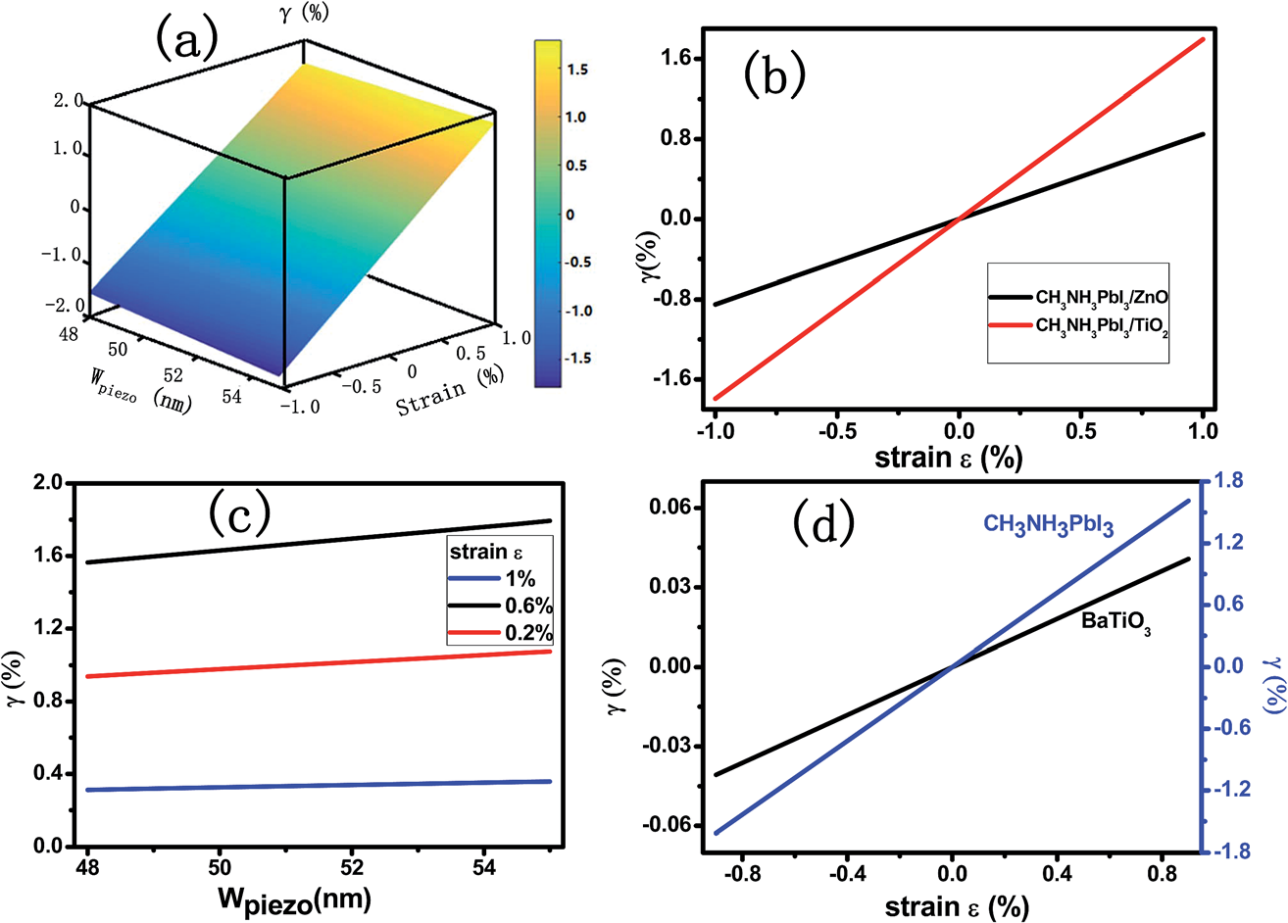
(a) The ratio *γ* of PPSC varies with *γ* and strain. (b) The ratio *γ* of PPSC increases with strain from −1% to 1% while *J*_solar_ is 14 mA cm^−2^ and 15.9 mA cm^−2^. (c) The control ratio *γ* of PPSC as a function of *W*_piezo_ with strains as 0.2%, 0.6% and 1% respectively. (d) The control ratio *γ* of PPSC changes with strain based on different perovskite materials: CH_3_NH_3_PbI_3_ and BATiO_3_.

**Fig. 4:**
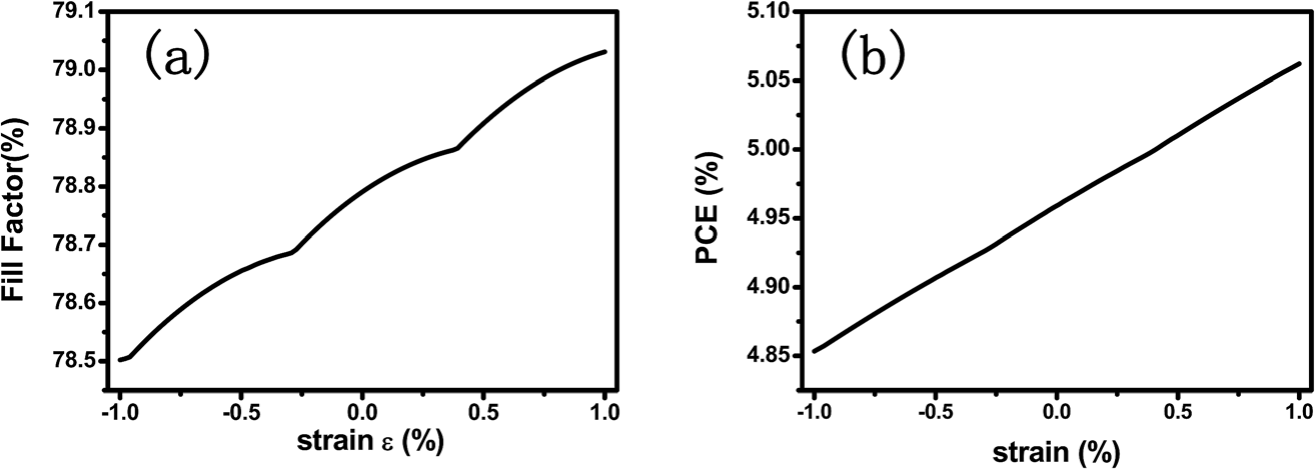
(a) Fill factor and (b) power conversion efficiency of PPSC with strain ranging from −1% to 1%.

The modulation ratio *γ* of PPSC in relation to *W*_piezo_ and strain *ε* is shown in Fig. 3(a). When *W*_piezo_ increases from 48 nm at −1.0% compressive strain to 55 nm at 1.0% tensile strain, the ratio *γ* varies from −1.56% to 1.79%. While an external strain is applied in the CH_3_NH_3_PbI_3_ layer, the performance of the PPSC improves linearly. Fig. 3(b) is the *γ* as a function of strain when *J*_solar_ is 14 mA cm^−2^ and 15.9 mA cm^−2^.^29^ The modulation ratio *γ* increases with the width of piezo-charge distribution *W*_piezo_, as illustrated in Fig. 3(c). Fig. 3(d) presents that the ratio *γ* as a function of the applied strain for two different piezoelectric perovskite materials: CH_3_NH_3_PbI_3_, BaTiO_3_. The piezoelectric constants for these two materials are 0.83 C m^−2^ (CH_3_NH_3_PbI_3_), 6.3 C m^−2^ (BaTiO_3_),^30^ respectively. The relative dielectric constants are 32 (CH_3_NH_3_PbI_3_), 600 (BaTiO_3_),^31^ respectively. Fig. 3(d) shows that the *γ* of CH_3_NH_3_PbI_3_ increases more significantly than that of BaTiO_3_ with strain varying from −1% to 1%. Though the piezoelectric constant *e*_33_ of BaTiO_3_ is much higher than that of the CH_3_NH_3_PbI_3,_ the regulation ability is restricted because of its extremely high dielectric constant. Using the eqn (4) and eqn (5), FF and PCE, two parameters describing the performance of PSCs, are presented in Fig. 4(a) and (b) with the externally applied strain varying from −1% to 1%. Fill factor and power conversion efficiency of CH_3_NH_3_PbI_3_ piezo-phototronic solar cell are enhanced due to increasing maximum output power and open circuit voltage with the external strain applied.

## 4. Conclusion

In summary, the theoretical model of piezo-phototronic perovskite solar cells is presented and subsequently calculated in this paper. The open circuit voltage, maximum output power, fill factor and power efficiency are calculated and analyzed. Piezo-phototronic perovskite solar cells have enhanced performance under applied strains. Furthermore, compared to other piezoelectric materials, CH_3_NH_3_PbI_3_ demonstrates superior potential for applications in high-efficiency piezo-phototronic solar cells for its relatively high modulation factor and easier fabrication process. The theoretic results deepen the physical understanding of the piezo-phototronic perovskite solar cells and provide future guidance for the design of organic photovoltaic nanodevices.

## Conflicts of interest

There are no conflicts to declare.

## Supplementary Material
